# Central and Peripheral Circulation Differ during Off-Pump Coronary Artery Bypass Grafting

**DOI:** 10.31083/j.rcm2501030

**Published:** 2024-01-16

**Authors:** Lars Saemann, Alina Zubarevich, Folker Wenzel, Jasmin Soethoff, Sevil Korkmaz-Icöz, Fabio Hoorn, Matthias Karck, Andreas Simm, Gábor Szabó, Gábor Veres

**Affiliations:** ^1^Department of Cardiac Surgery, University of Halle, 06120 Halle, Germany; ^2^Department of Cardiac Surgery, University of Heidelberg, 69120 Heidelberg, Germany; ^3^Department of Cardiac-, Thoracic-, Transplantation and Vascular Surgery, Hannover Medical School, 30625 Hannover, Germany; ^4^Faculty Medical and Life Sciences, Furtwangen University, 78054 Villingen-Schwenningen, Germany

**Keywords:** CABG, OPCAB, perfusion, microcirculation, cutaneous oxygen partial pressure

## Abstract

**Background:**

Off-pump coronary artery bypass grafting (OPCAB) is an alternative to on-pump coronary artery bypass grafting (CABG) with cardiopulmonary bypass (CPB). During OPCAB, the temporary use of an intracoronary shunt and inotropic medication or catecholamines should keep the central hemodynamics constant. Nevertheless, the need for conversion to on-pump CABG often occurs unexpectedly, most likely due to circulation instability. Circulation instability can appear first in peripheral body parts; therefore, peripheral microcirculation might serve as a predictor for the upcoming conversion to on-pump CABG. We investigated the impact of coronary artery ligation and shunt insertion during OPCAB on cutaneous microcirculation (cLDP) with Laser Doppler Perfusion Technology and transcutaneous oxygen partial pressure (${\mathrm{tcpO}}_{2}$).

**Methods:**

In a pig model of OPCAB, peripheral circulation was evaluated after cLDP (N = 17) and ${\mathrm{tcpO}}_{2}$ (N = 6) monitoring. Systolic, diastolic, and mean arterial pressure were also observed to prove the independence of perfusion measurement results from hemodynamic parameters.

**Results:**

Ligation time during cLDP and ${\mathrm{tcpO}}_{2}$ monitoring were 101 ± 49 s and 83 ± 33 s, respectively. Shunt time was 11 ± 3 min during cLDP and 13 ± 2 min during ${\mathrm{tcpO}}_{2}$ measurement. Ligation of the left anterior descending coronary artery (LAD) reduced cLDP significantly to 88 ± 14% (*p* = 0.007) and ${\mathrm{tcpO}}_{2}$ to 71 ± 25% (*p* = 0.038). Inserting a temporary shunt into the LAD significantly improved cLDP (*p* = 0.006) and ${\mathrm{tcpO}}_{2}$ (*p* = 0.015) compared to ligation. cLDP was restored to 99%, and ${\mathrm{tcpO}}_{2}$ was restored to 91% of the baseline level before ligation. All hemodynamic parameters remained stable and did not change significantly during OPCAB.

**Conclusions:**

Although hemodynamic parameters stayed constant, peripheral microcirculation was influenced markedly during OPCAB. Inserting a temporary shut into the LAD leads to a complete normalization of peripheral microcirculation, regarding evaluation by cLDP and ${\mathrm{tcpO}}_{2}$.

## 1. Introduction

Coronary artery bypass grafting (CABG) is the method of choice for the surgical treatment of coronary heart disease. CABG can either be performed on the cardioplegic heart while cardiopulmonary bypass (CPB) maintains the circulation of the body, known as on-pump CABG, or on the beating heart without CPB, known as off-pump CABG (OPCAB). OPCAB is applied to avoid potential adverse effects from the cardioplegic heart and CPB, such as ischemia/reperfusion injury, coagulation activation, systemic inflammation, endothelial dysfunction, and oxidative stress [1]. Although OPCAB rarely needs to be converted to on-pump CABG, a low rate of conversions still exists. The most common reason for conversion to on-pump is hemodynamic instability. Surgery-associated reasons for conversion, such as difficult anastomozation because of small vessels or inadequate visualization, are less frequent [2]. However, small vessels make anastomozation very complicated.

During OPCAB, inserting an intracoronary shunt instead of ligating the coronary artery during anastomozation reduces myocardial dysfunction [3]. Nevertheless, administering inotropic medication according to the typical dynamic hemodynamic demands during OPCAB remains necessary. Despite the use of intracoronary shunts, inotropes, or catecholamines, the necessity to convert OPCAB to on-pump CABG often occurs spontaneously.

The circulation is only monitored by central, macrovascular hemodynamics. Nevertheless, the global circulation of the body might behave inconsistently. Thus, we hypothesize that even under consistent hemodynamics due to coronary shunts and inotropes or catecholamines, tissue perfusion is not stable in the whole body during OPCAB.

Circulation instability can often be observed first in peripheral body parts before central hemodynamics change. It might, therefore, be of high interest as a measure for early prediction for conversion to on-pump. Considering all aspects, this work investigates the course of central hemodynamics and peripheral microcirculation to evaluate circulation instability during OPCAB.

## 2. Material and Methods

### 2.1 Animal Preparation and Anesthesia

In an experimental model of CABG surgery, healthy pigs with a body weight between 45 and 55 kg underwent OPCAB surgery. The local Ethical Committee for Animal Experimentation (Regierungspräsendium Karlsruhe) reviewed and approved the investigations. Anesthesia was induced with Ketamine and Midazolam. After intubation was performed, anesthesia was maintained intravenously with Propofol 2% (B. Braun, Melsungen, Germany). During the surgical procedure on the beating heart, norepinephrine (Arterenol, Sanofi-Aventis Deutschland GmbH, Frankfurt am Main, Germany) was infused to keep arterial blood pressure stable if necessary.

### 2.2 Surgical Technique

**Fig. 1.**
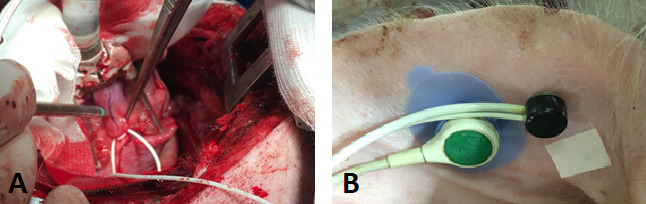
**Animal operation**. (A) Ligating the left anterior descending coronary artery. (B) Probes. Black probe: Measurement of Laser Doppler Perfusion. Green probe: Measurement of transcutaneous oxygen partial pressure.

A median sternotomy followed by pericardiotomy was performed. Internal thoracic arteries were harvested, and heparin was administered to avoid coagulation in the bypass vessels. The left anterior descending coronary artery (LAD) was exposed and ligated so that bleeding could have been avoided during the insertion of an intracoronary shunt (Fig. 1). While the shunt re-established the blood flow through the LAD, the graft was anastomosed.

### 2.3 Monitoring of Peripheral Perfusion

We have already shown that Laser Doppler Perfusion Monitoring (LDPM) is a very sensitive method for observingffective tissue perfusion on the microcirculatory level in cardiac surgical patients [4]. In LDPM, the measured data is proportional to microcirculation and expressed in relative units, compared to a baseline measurement [5]. We applied two monitoring methods for peripheral perfusion: LDPM to measure cutaneous microcirculation (cLDP) in 17 subjects and measurement of transcutaneous oxygen partial pressure (${\mathrm{tcpO}}_{2}$) in 6 subjects. Probes (Laser Doppler Small Angeled Probe 547; ${\mathrm{tcpO}}_{2}$ Probe; Perimed, Järfälla-Stockholm, Sweden) were placed on the inside of the left ear (Fig. 1). The skin was prepared according to the manufacturer’s instructions. The time constant of the Laser Doppler Perfusion (LDP) probe was 0.2 s to achieve a sampling rate of 5 Hz. Furthermore, the probe was characterized by a laser wavelength of 780 nm.

### 2.4 Statistical Analysis

Statistical analysis was performed using IBM SPSS Statistics for Windows (Version 20.0, IBM Corp., Armonk, NY, USA). All measurement values are presented as mean ± standard error. A repeated measures one-way analysis of variance with Bonferroni adjusted comparison of main effects was executed for cLDP and all hemodynamic comparisons. For ${\mathrm{tcpO}}_{2}$ comparison, a repeated measures one-way analysis of variance with Least Significant Difference (LSD) adjusted comparison of main effects was performed to address the relatively low number of subjects. A *T*-test was executed for the comparison of ligation time and shunt time. A value of *p* < 0.05 was considered statistically significant.

## 3. Results

**Fig. 2.**
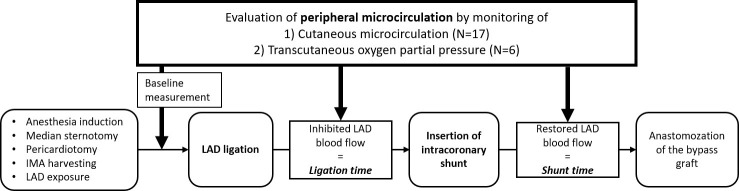
**Flow chart of the measurement sequence**. IMA, internal mammary artery; LAD, left anterior descending coronary artery.

Peripheral microcirculation was monitored before LAD ligation, after LAD ligation, and after inserting an intracoronary shunt (Fig. 2). Both cLDP and ${\mathrm{tcpO}}_{2}$ are typically expressed in relative units and designated as relative cLDP and ${\mathrm{tcpO}}_{2}$ [5].

### 3.1 Peripheral Microcirculation

**Fig. 3.**
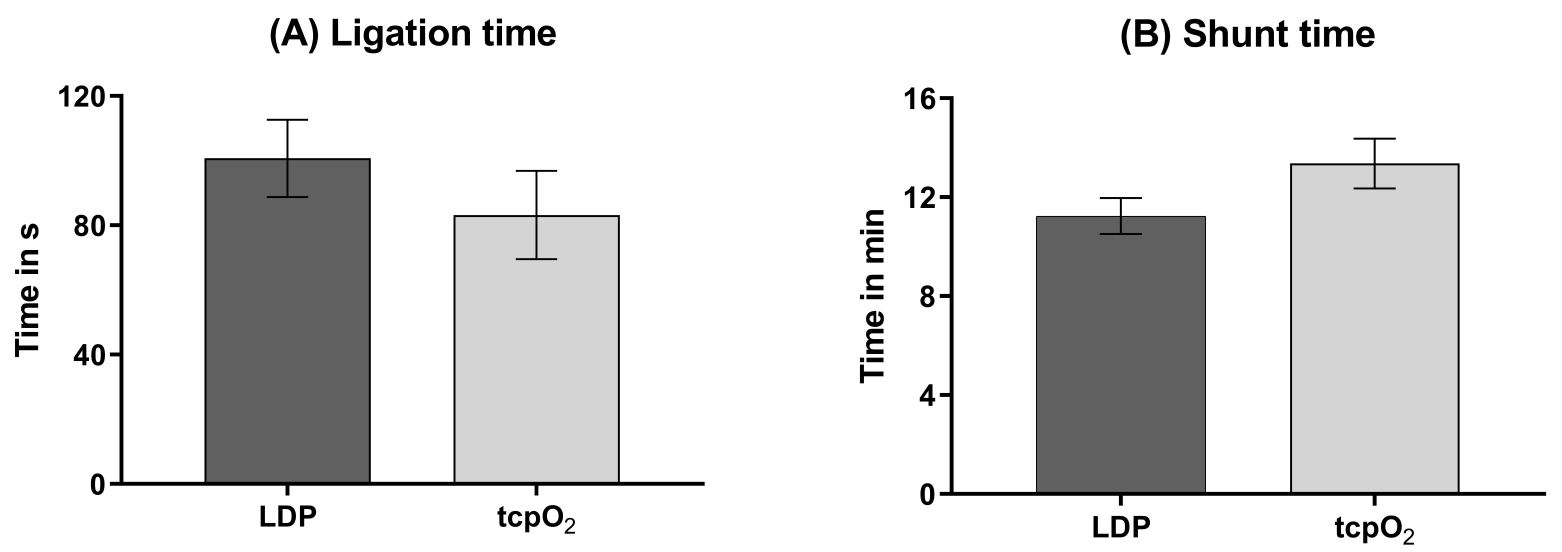
**Critical periods**. (A) Ligation time. (B) Shunt time. N (LDP) = 17. N (${\mathrm{tcpO}}_{2}$) = 6. LDP, Laser Doppler Perfusion; ${\mathrm{tcpO}}_{2}$, transcutaneous oxygen partial pressure; N, number of animals.

**Fig. 4.**
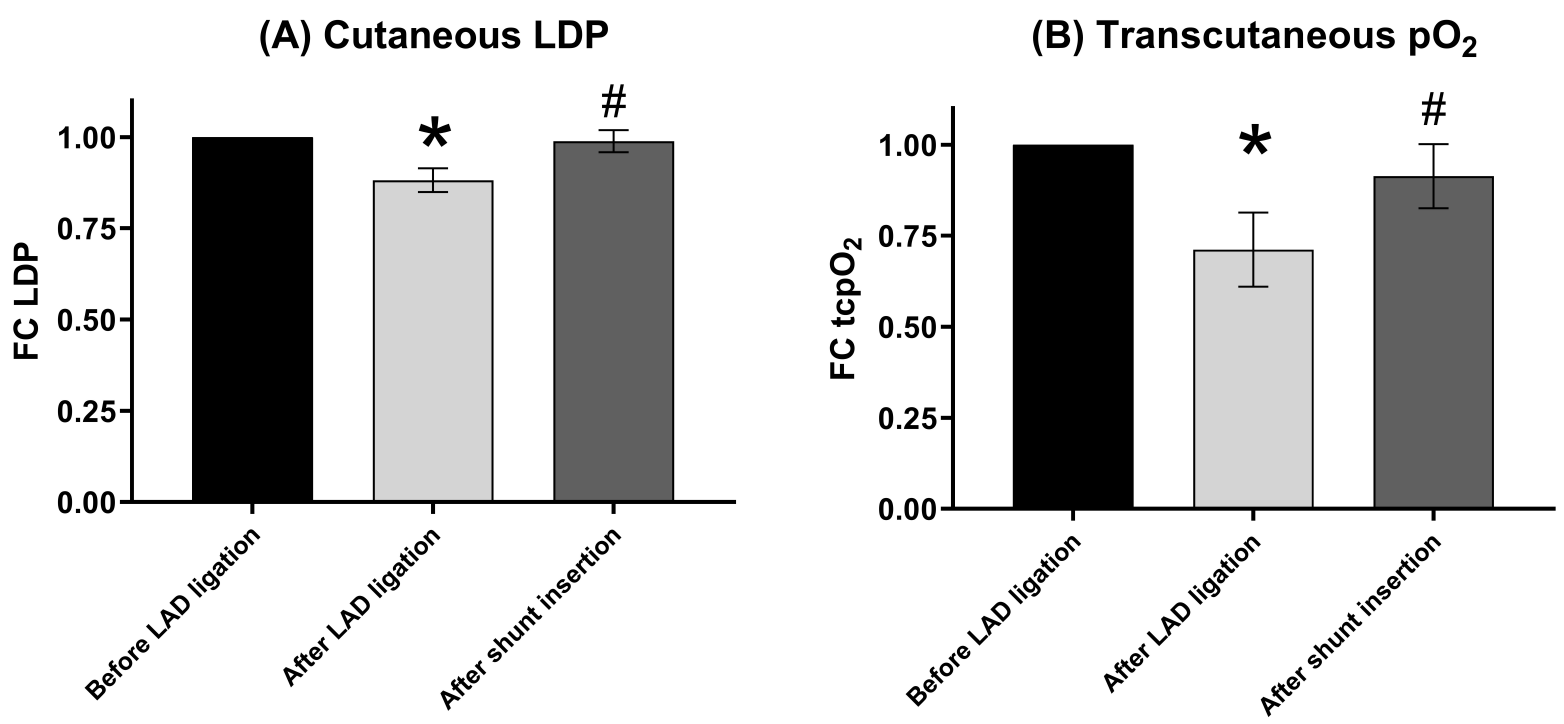
**Peripheral microcirculation during OPCAB**. (A) Cutaneous LDP. (B) Transcutaneous ${\mathrm{pO}}_{2}$. Bars with error bars represent mean ± standard deviation. *p < 0.05 compared to baseline measurement before LAD ligation. ^#^*p* < 0.05 compared to measurement after LAD ligation. N (LDP) = 17. N (${\mathrm{tcpO}}_{2}$) = 6. FC, fold change; LAD, left anterior descending coronary artery; LDP, Laser Doppler Perfusion, proportional to microcirculation; ${\mathrm{tcpO}}_{2}$, transcutaneous oxygen partial pressure; OPCAB, off-pump coronary artery bypass grafting; N, number of animals.

LAD blood flow was inhibited by ligation for 101 ± 49 s during cLDP and 83 ± 33 s during ${\mathrm{tcpO}}_{2}$ measurements (Fig. 3). The mean shunt time during cLDP measurement was 11 ± 3 min and 13 ± 2 min during the ${\mathrm{tcpO}}_{2}$ measurement (Fig. 3). Ligation of the LAD (Fig. 4) resulted in a significant reduction of cLDP by about 12% (*p* = 0.007; 95% CI 0.031, 0.208). ${\mathrm{tcpO}}_{2}$ was reduced even more drastically by about 19% (*p* = 0.038; 95% CI 0.024, 0.549). After shunt insertion, cLDP was stabilized to 99% of baseline level (*p* = 1.000; 95% CI –0.069, 0.092) and significantly higher than total LAD ligation (*p* = 0.006; 95% CI –0.186, –0.029). The highest ${\mathrm{tcpO}}_{2}$ was observed at the end of shunt time at 91% of baseline level (*p* = 0.371; 95% CI –0.139, 0.311) and significantly improved compared to LAD ligation (*p* = 0.015; 95% CI –0.342, –0.060).

### 3.2 Hemodynamics

**Fig. 5.**
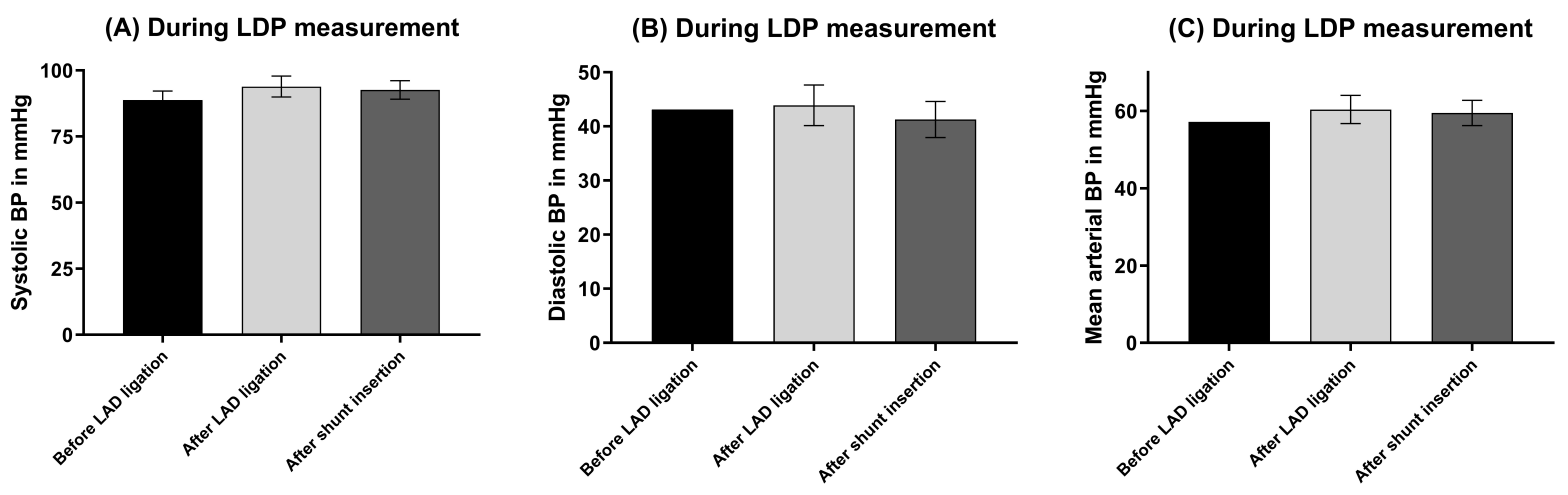
**Hemodynamics during LDP measurement**. (A) Systolic blood pressure. (B) Diastolic blood pressure. (C) Mean arterial blood pressure. No significant differences between groups. N = 16. BP, blood pressure; LDP, Laser Doppler Perfusion; LAD, left anterior descending coronary artery; N, number of animals.

**Fig. 6.**
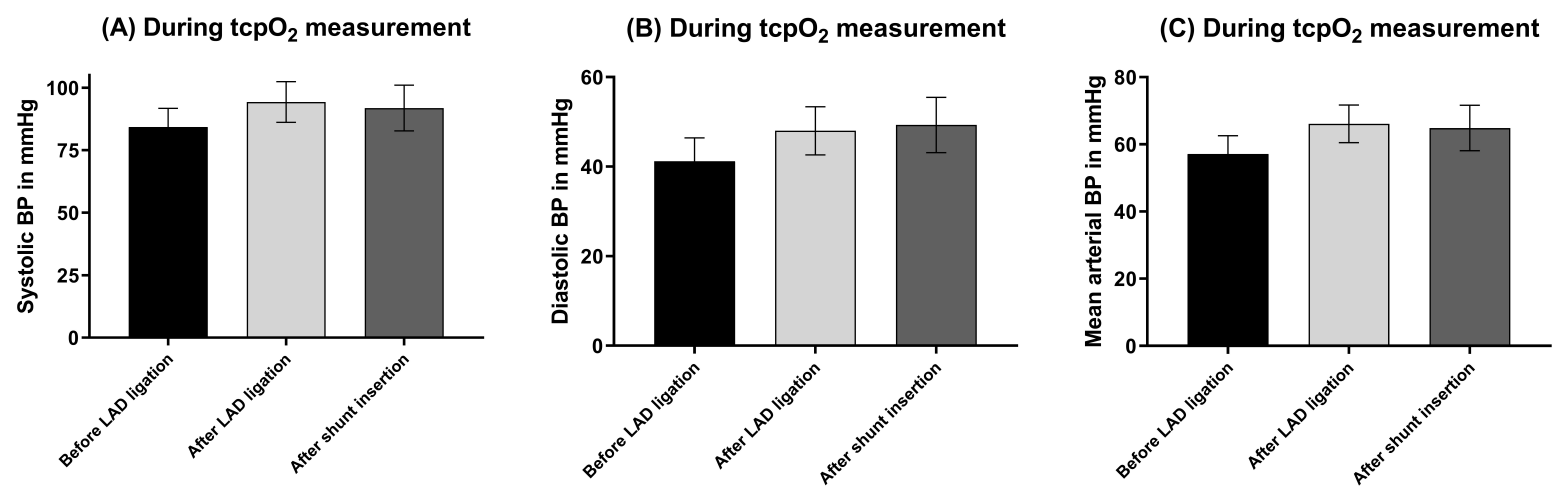
**Hemodynamics during ${\mathrm{tcpO}}_{2}$ measurement**. (A) Systolic blood pressure. (B) Diastolic blood pressure. (C) Mean arterial blood pressure. No significant differences between groups. N = 6. BP, blood pressure; LDP, Laser Doppler Perfusion; LAD, left anterior descending coronary artery; ${\mathrm{tcpO}}_{2}$, transcutaneous oxygen partial pressure; N, number of animals.

In one pig, hemodynamics (Fig. 4) could not have been monitored during cLDP measurement due to technical failure of the monitoring system. Systolic, diastolic, and mean arterial blood pressure did not significantly change throughout the evaluation period of peripheral perfusion by both cLDP (Fig. 5) and ${\mathrm{tcpO}}_{2}$ (Fig. 6) monitoring.

### 3.3 Catecholamine Administration

In the LDP group in N = 17 pigs (100%), no catecholamines were administered during the observation of peripheral perfusion. During ${\mathrm{tcpO}}_{2}$ administration in N = 1 pig (17%), norepinephrine was administered during the ligation of LAD. The infusion rate was kept constant while the shunt was inserted.

## 4. Discussion

Both measurement technologies indicated a significantly improved peripheral microcirculation by inserting a temporary shunt into the LAD. Total ligation of the LAD leads to temporal ischemia of the myocardium, as shown in a study based on surrogate parameters by Takami *et al*. [6]. Myocardial ischemia leads to myocardial dysfunction [3] and consequently to a decreased perfusion of the whole body. Arterial blood pressure was kept constant during LAD ligation by vasoconstrictive medication. This indicates that responsive and vigilant anesthesia management can keep hemodynamics constant during OPCAB.

Nevertheless, this does not prevent peripheral circulation instability and thus confirms our hypothesis that global circulation is not stable during OPCAB even under stable arterial blood pressure. In the present work, peripheral perfusion seems downregulated to maintain circulation in central body parts and the heart, resulting in partial peripheral malperfusion. As the insertion of an intracoronary shunt can at least partially re-establish the myocardial blood flow [6], circulation in the periphery was also re-established analogously. The reduced circulation led to an oxygen debt in peripheral body parts, as shown by ${\mathrm{tcpO}}_{2}$ monitoring. We assume that ${\mathrm{tcpO}}_{2}$ did not reach the baseline level. However, cLDP was fully re-established because oxygen debt in the periphery was not completely compensated until the end of the measurement.

Except for one pig, no catecholamine administration was necessary to keep blood pressure constant during ligation and shunt insertion. The propofol infusion was also not changed during the procedure. Therefore, we conclude that external catecholamine administration did not overlay the actual effects in the peripheral microcirculation, leading to an artificially reduced LDP and ${\mathrm{tcpO}}_{2}$.

Another important factor that might impact the results is the surgeon and the respective duration to finish the anastomoses. However, the same experienced cardiac surgeon performed all anastomosis during this project.

### 4.1 Measurement of Peripheral Microcirculation

Another method to monitor microvascular circulation, tested in cardiac surgical patients, is camera-based photoplethysmography [7]. This technology is less well established in a clinical setting, by far more space-consuming than LDP or ${\mathrm{tcpO}}_{2}$ monitoring, and cannot measure ${\mathrm{tcpO}}_{2}$. Thus, we refused to use this technology [8].

An alternative to the continuous observation of LDP is moderate local heating of the skin by special thermostatic LDP probes to verify the thermal reactivity of cutaneous microvasculature [9]. A third but rarely applied method is investigating the local effect of acetylcholine or sodium nitroprusside on cutaneous microcirculation [10]. Both heat and pharmacological activation demand a more extended period to build up an effect or subside than the ligation time or shunt time. Consequently, they are inapplicable to evaluating cutaneous perfusion during OPCAB.

A permanent monitoring of LDP and ${\mathrm{tcpO}}_{2}$ creates a curve of measured data. A few studies report the analysis of LDP and ${\mathrm{tcpO}}_{2}$ by calculating the area under the curve (AUC) rather than analyzing measurement data itself. Based on our own experience, which is fully confirmed by the results from Salgado *et al*. [9], analysis of AUC is less sensitive than calculating the relative change of data between baseline and intervention measurement.

As known from a clinical setting of LDPM on patients, cutaneous microcirculation is measured on the finger [4], forearm [9], forehead [9, 11], or foot [12]. These areas of the skin appear different and tougher in a pig already by macroscopic inspection compared to human skin. Therefore, we decided to choose the inside of the left ear to place probes. All medication was administered through a vein of the other ear to prevent the immediate effect on local microvascular circulation, which could have occurred from anesthetics, analgesics, or vasoconstrictive agents.

### 4.2 Future Perspectives

Monitoring cLDP and ${\mathrm{tcpO}}_{2}$ is easy and safe to establish and does not influence the operation. Both probes are approved to be used on patients, so verifying our results by monitoring patients would be simple to perform. Furthermore, we consider if our monitoring methods of cutaneous circulation can serve as an early detection model for the upcoming conversion from OPCAB to on-pump CABG, as it can occur unexpectedly.

### 4.3 Limitations

A larger series on ${\mathrm{tcpO}}_{2}$ monitoring during OPCAB would be of high interest. Second, cLDP and ${\mathrm{tcpO}}_{2}$ monitoring are local observations of peripheral microcirculation. LDP imagers could be of potential benefit to monitoring microcirculation in large areas. Another limitation is that we applied anatomozation only on the LAD. Anastomozation on other coronary arteries, such as the circumflex artery [13], and the effects on peripheral and central circulation should also be investigated.

## 5. Conclusions

Evaluation of peripheral microcirculation by cLDP and ${\mathrm{tcpO}}_{2}$ monitoring leads to a deeper understanding of the regulation of peripheral microcirculation during OPCAB surgery with an intracoronary shunt. Even when central hemodynamics stay constant, peripheral microcirculation is not stable. An intracoronary shunt not only restores myocardial perfusion, as shown by Takami *et al*. [6], but also peripheral microcirculation, as shown by our results. Furthermore, we conclude that LDP and ${\mathrm{tcpO}}_{2}$ measurements are safe, easy to perform, and facilitate sensitive methods to monitor peripheral microcirculation.

## Data Availability

The datasets used and/or analyzed during the current study are available from the corresponding author on reasonable request.

## Abbreviations

AUC, area under the curve; CABG, coronary artery bypass grafting; cLDP, cutaneous microcirculation; CPB, cardiopulmonary bypass; LAD, left anterior descending coronary artery; LDPM, Laser Doppler Perfusion Monitoring; OPCAB, off-pump coronary artery bypass grafting; r-cLDP, relative cutaneous Laser Doppler Perfusion; ${\mathrm{r-tcpO}}_{2}$, relative transcutaneous oxygen partial pressure; ${\mathrm{tcpO}}_{2}$, transcutaneous oxygen partial pressure.
